# Supplementary material for: Classifying seismograms using the FastMap algorithm and support-vector machines
Source: Commun Eng. 2023 Jul 15;2:46. doi: 10.1038/s44172-023-00099-8 (PMC10955874; doi:10.1038/s44172-023-00099-8)
Supplement: Supplementary file 2 — Supplemental Material [file 44172_2023_99_MOESM2_ESM.pdf]

# Supplementary Material for “Classifying Seismograms Using the FastMap Algorithm and Support-Vector Machines” (White et al., 2023)

Supplementary Figures S1 and S2.

Supplementary Text S1 (Hand-Written Digit Classification via FastMapSVM).

Supplementary Figures S3 and S4.

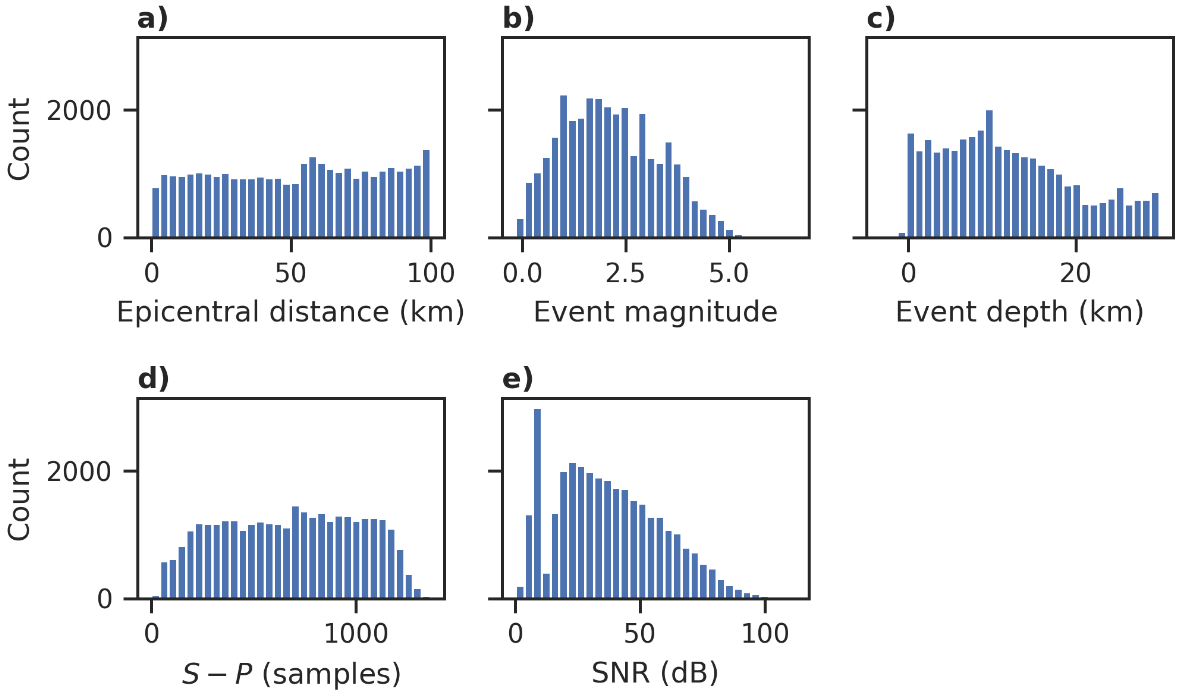


**Fig. S1. Distribution of earthquake seismograms sampled from STEAD.** Shows the distribution of various features of earthquake seismograms represented in the combined train and test data set sampled from STEAD. (a), (b), (c), (d), and (e) show the distribution of epicentral distances, event magnitudes, event depths, the intervals between P- and S-wave arrivals, and SNRs, respectively. The distributions represent 32 768 earthquake seismograms; noise seismograms are excluded as these features are non-existent.

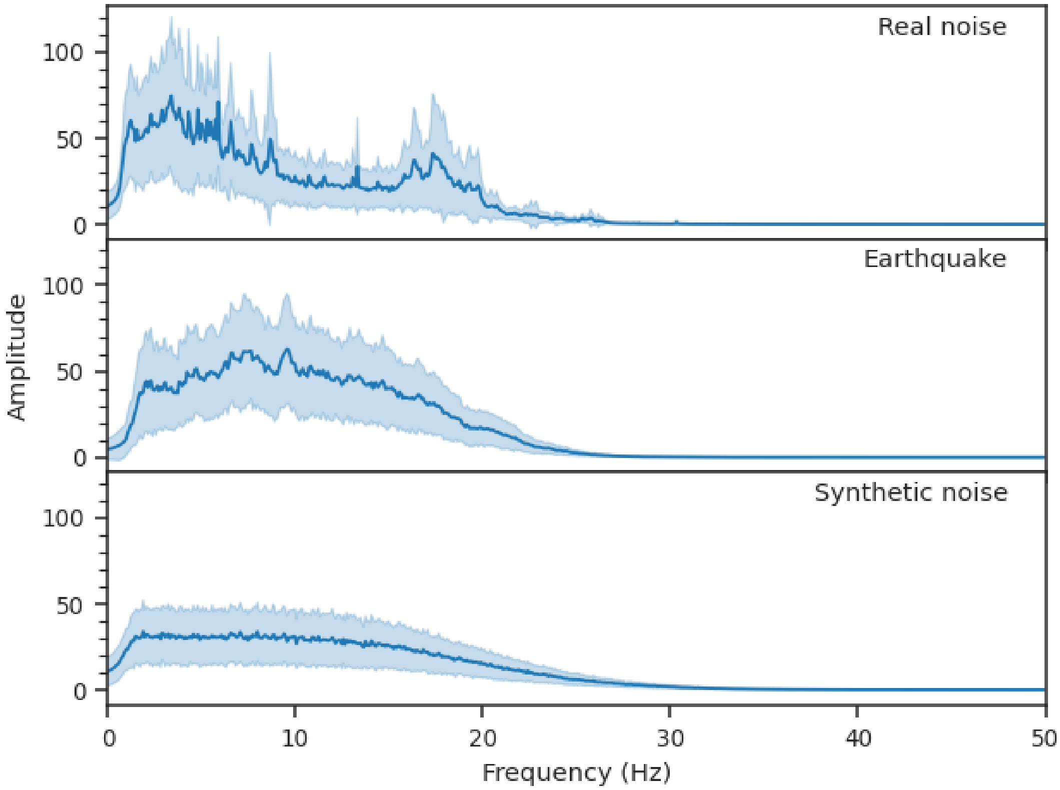


**Fig. S2. Average seismogram frequency spectra.** Shows the typical frequency spectra of real noise, earthquake signals, and added synthetic noise.

# Supplementary Text S1

**Hand-Written Digit Classification via FastMapSVM.** In a supplementary application that demonstrates the viability of FastMapSVM, we use it to classify images of hand-written digits from the MNIST (Modified National Institute of Standards and Technology) database, a standard benchmark data set for testing image classification algorithms.

The train and test sets of the MNIST database comprise 60 000 and 10 000 instances, respectively, in which each instance is a $28 \times 28$ pixels, grey-scale image of a hand-written digit between '0' and '9'. We use the train and test sets from the MNIST database without any alterations. We train one FastMapSVM classifier for each digit in a "one-versus-rest" framework: For a given digit, all instances of that digit are labeled positive, and all other instances are labeled negative. The classifiers are trained to predict the probability of a test instance being positive. The FastMapSVM classifiers use 1 minus the cosine similarity function as the distance function between the images. In the testing phase, the predicted class of a test instance is the positive class returned with maximum probability by any classifier.

Even with the simple distance function mentioned above, FastMapSVM achieves a high classification accuracy of 98.03 % (Figs. S3 and S4).

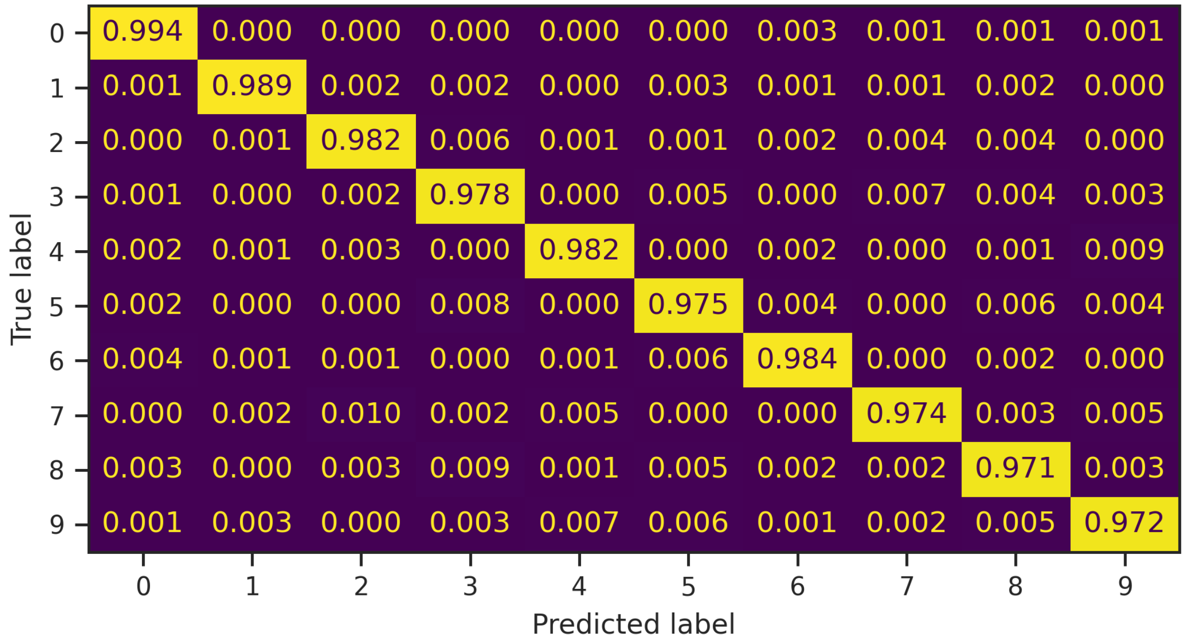


**Fig. S3. Confusion matrix for FastMapSVM's classification results on the MNIST hand-written digits database.** Shows the confusion matrix for the classification results obtained by FastMapSVM on the MNIST hand-written digits database.

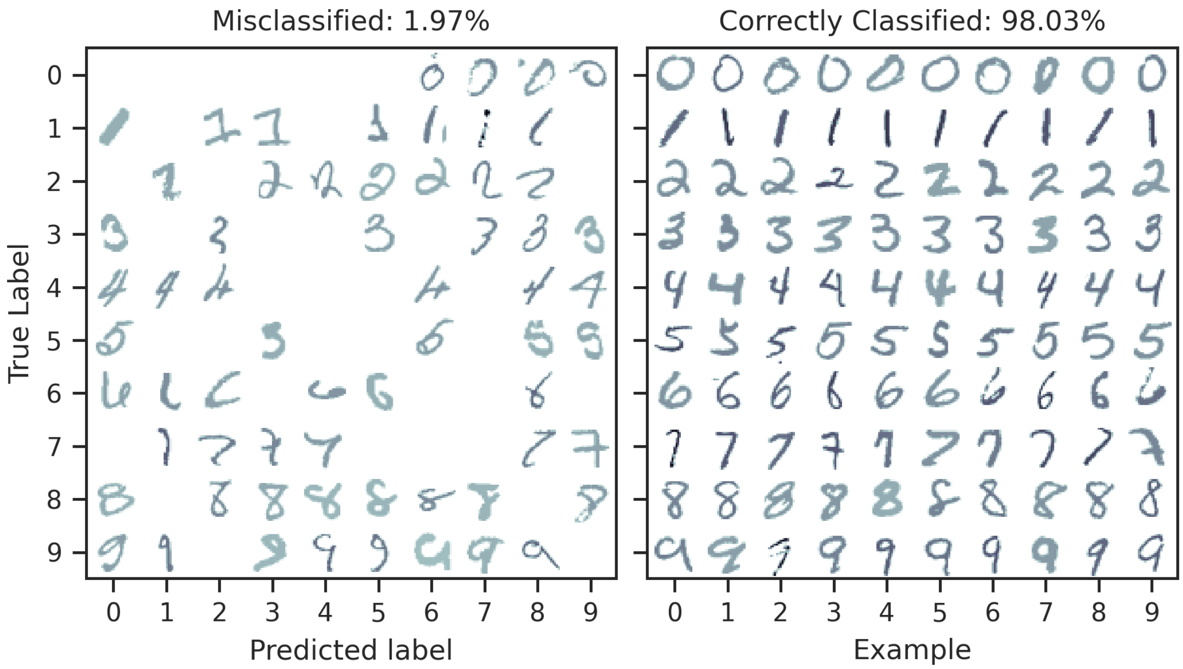


**Fig. S4. Examples of FastMapSVM's misclassified and correctly classified test instances from the MNIST hand-written digits database.** Shows examples of test instances from the MNIST hand-written digits database that are misclassified and correctly classified by FastMapSVM. (a) shows the misclassified examples with their true label on the vertical axis and their predicted label on the horizontal axis. (b) shows $10$ correctly classified examples for each digit with their true label on the vertical axis.
